# Association of the TyG index and inflammatory burden index with AMR-defined coronary microvascular dysfunction and 12-month MACE after STEMI

**DOI:** 10.3389/fendo.2026.1900132

**Published:** 2026-08-07

**Authors:** Yang Yang, Leilei Liu, Xiuyu Liang, Xiaohong Zhang, Hai Xu

**Affiliations:** Department of Cardiology, The Third Affiliated Hospital of Anhui Medical University (The First People’s Hospital of Hefei), Hefei, Anhui, China

**Keywords:** angiography-derived microvascular resistance, coronary microvascular dysfunction, inflammatory burden index, prognosis, triglyceride-glucose index

## Abstract

**Background:**

Metabolic dysfunction and inflammation may jointly contribute to coronary microvascular injury and adverse outcomes after primary percutaneous coronary intervention (PPCI) for ST-segment elevation myocardial infarction (STEMI). We examined the independent and combined associations of the triglyceride-glucose (TyG) index and inflammatory burden index (IBI) with angiographic microvascular resistance (AMR)-defined coronary microvascular dysfunction (CMD), their incremental value beyond conventional factors, and their associations with 12-month major adverse cardiovascular events (MACE).

**Methods:**

In this retrospective cohort study conducted at a single center, 318 STEMI patients who underwent PPCI and achieved a TIMI grade 3 flow post-procedure were enrolled. AMR was derived from the final post-PPCI angiogram of the culprit vessel, and CMD was defined as AMR >26.6 mmHg·s/dm. Multivariable models assessed associations with CMD and MACE. Sequential models evaluated the incremental performance of TyG and IBI with 1,000-resample bootstrap internal validation.

**Results:**

CMD was present in 102/318 (32.1%). TyG (OR per 1-unit, 3.83; 95% CI, 2.04–7.21; P<0.001) and IBI (OR per 10-units, 1.13; 95% CI, 1.07–1.20; P<0.001) were independently associated with CMD. The TyG–IBI model showed an AUC of 0.773 for CMD; adding both to a clinical/procedural model improved the AUC from 0.904 to 0.939 (bootstrap-validated, 0.920; ΔAUC, 0.036; P = 0.001). Over 12 months, 69 (21.7%) patients had MACE. TyG (HR, 1.97; 95% CI, 1.30–2.98; P = 0.001) and IBI (HR per 10-units, 1.02; 95% CI, 1.00–1.05; P = 0.022) remained independent predictors of MACE, with the highest risk in those with both markers elevated.

**Conclusion:**

TyG and IBI were independently associated with AMR-defined CMD and 12-month MACE after STEMI. Adding TyG and IBI to conventional clinical and procedural factors improved model discrimination for CMD, although prospective multicenter validation is required before clinical application.

## Introduction

Acute STEMI, a severe clinical presentation of acute myocardial infarction, significantly contributes to high patient morbidity and mortality (1). Although PPCI effectively restores epicardial coronary artery blood flow, myocardial tissue perfusion remains inadequate in some patients even after successful epicardial reperfusion (2). CMD, including microvascular obstruction and the no-reflow phenomenon, may persist despite final Thrombolysis in Myocardial Infarction (TIMI) grade 3 flow and is associated with larger infarct size, impaired ventricular recovery, and adverse clinical outcomes (2–4). Recent studies demonstrating the prognostic relevance of incomplete ST-segment resolution and treatment delays further emphasize that epicardial patency alone does not fully reflect myocardial reperfusion after PPCI (5, 6). Therefore, reliable identification of residual microvascular injury remains clinically relevant in patients with STEMI.

The conventional intraoperative assessment method for CMD, the index of microvascular resistance (IMR), is challenging to incorporate routinely into emergency PPCI due to the requirement for pressure wires and vasodilators like adenosine (7). Recently, μQFR technology, employing single-view Murray’s law principles, has introduced AMR, a novel parameter to evaluate microcirculation. AMR does not necessitate pressure wires or vasodilators and has shown robust correlation with invasive IMR (8–10). The multicenter EARLY-MYO-AMR study demonstrated that an AMR threshold greater than 26.6 mmHg·s/dm accurately identifies microvascular dysfunction and predicts a higher incidence of MACE (9). Consequently, AMR shows promise as a feasible catheterization-laboratory method for diagnosing CMD and conducting early risk assessments in STEMI cases.

IR serves as a vital marker of metabolic dysfunction, significantly affecting the pathophysiology of cardiovascular metabolic diseases, particularly by disrupting myocardial perfusion and the function of coronary microvasculature in patients experiencing STEMI (11, 12). The TyG index, derived from fasting glucose and triglyceride levels, serves as a practical and dependable indicator for assessing IR (13). Higher TyG levels have also been associated with the coronary slow-flow phenomenon (14). Söner et al. further reported that a higher TyG index was associated with an increased risk of contrast-induced nephropathy among patients undergoing PCI for chronic total occlusion (15). Nonetheless, the evidence directly linking the TyG index to coronary microvascular dysfunction, particularly AMR-defined CMD in patients with STEMI, remains limited. Concurrently, inflammation serves as a crucial mechanism that contributes to ischemia-reperfusion injury and the ensuing microvascular dysfunction observed in STEMI (16). The IBI, a composite inflammatory marker integrating C-reactive protein and the neutrophil-to-lymphocyte ratio, has been associated with cardiovascular disease, coronary slow flow, and outcomes in acute decompensated heart failure (17–19) Nevertheless, data are still limited regarding whether IBI is directly associated with acute-phase CMD in STEMI and whether a combined metabolic-inflammatory assessment using TyG and IBI may improve prediction of microvascular injury and long-term outcomes.

Previous evidence suggests that combining metabolic and inflammatory markers may improve cardiovascular risk stratification after STEMI (20). We hypothesized that TyG and IBI provide complementary information on coronary microvascular injury. This study therefore evaluated their independent and combined associations with culprit-vessel AMR-defined CMD, their incremental discrimination beyond conventional factors, and their associations with 12-month MACE.

## Methods

### Study design and participants

This observational cohort study was conducted retrospectively at a single institution. Patients consecutively admitted for acute STEMI undergoing PPCI at the Cardiology Department of Anhui Medical University’s Third Affiliated Hospital from June 2021 through June 2024 were enrolled. STEMI was diagnosed according to the Fourth Universal Definition of Myocardial Infarction and contemporary guidelines for acute coronary syndromes (21, 22). Criteria for inclusion consisted of (1): undergoing PPCI within 12 hours from symptom onset (2); successful re-establishment of TIMI grade 3 flow in the culprit vessel after intervention. Exclusion criteria consisted of (1): concurrent severe structural cardiac diseases, including dilated cardiomyopathy, moderate-to-severe valvular disorders (aortic or mitral valve stenosis/regurgitation), or congenital ventricular septal defects (2); failure to administer dual antiplatelet therapy post-PPCI (3); severe infections, active autoimmune conditions, or malignancies (4); significant hepatic or renal impairment [eGFR < 30 mL/min/1.73 m^2^ or Child-Pugh class C] (5); suboptimal angiographic images or absence of essential angiographic projections impeding precise AMR evaluation; and (6) incomplete baseline clinical information or inadequate follow-up data.

**Figure 1:**
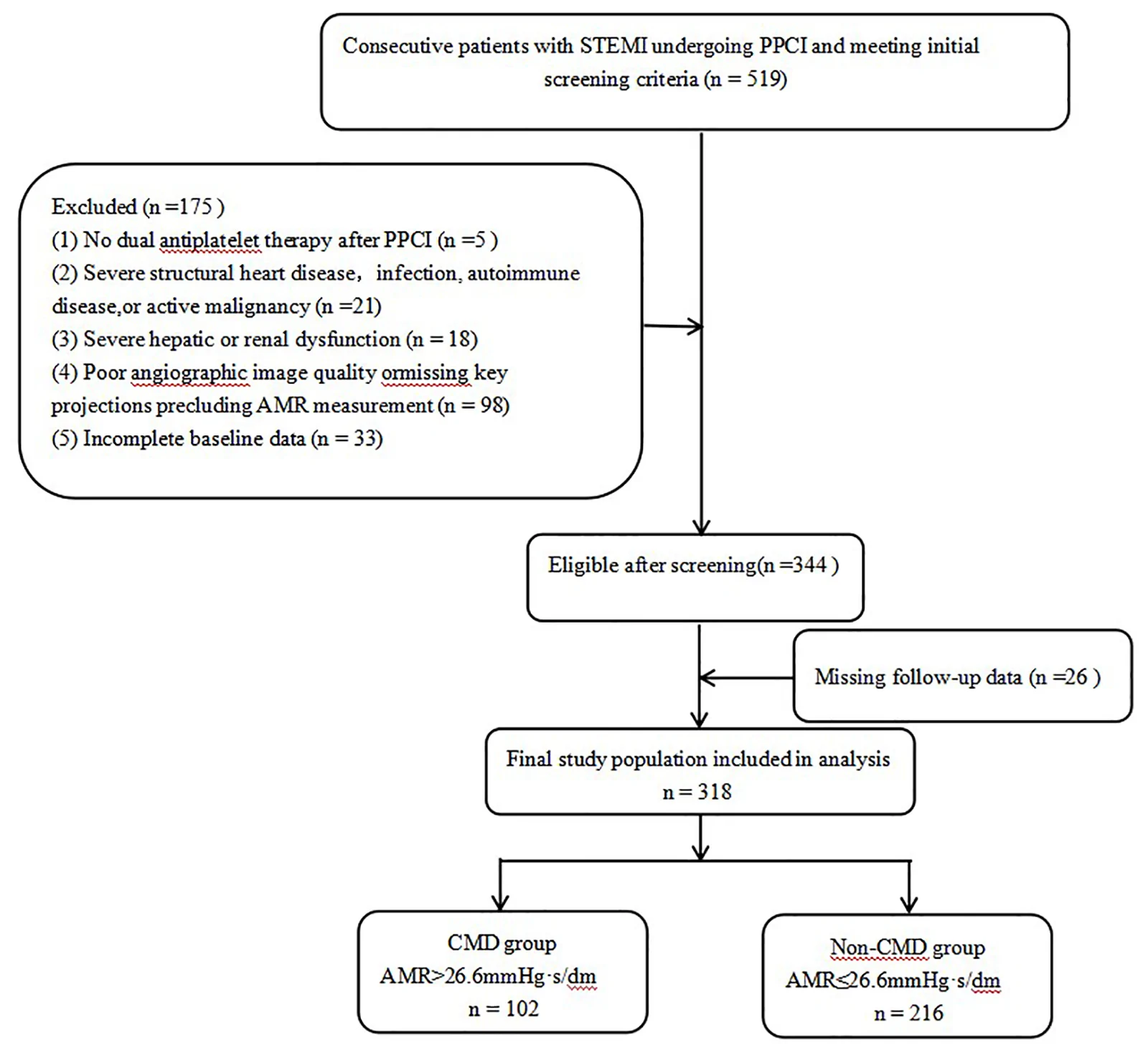
Study flowchart.

Of 519 initially screened patients, 175 were excluded during baseline screening, including 33 (6.4%) because of incomplete baseline data. Among 344 otherwise eligible patients, 26 (7.6%) were excluded because adequate follow-up information was unavailable. The final complete-case cohort comprised 318 patients with complete data for variables included in the primary logistic and Cox models (**Figure 1**). The study complied with the Declaration of Helsinki and was approved by the Ethics Committee of the Third Affiliated Hospital of Anhui Medical University (Approval No. 2025-037). Because of the retrospective design, informed consent was waived.

### Data collection

Baseline characteristics were meticulously gathered from electronic medical records, laboratory information databases, and catheterization laboratory documentation, including demographic information (age, gender), cardiovascular risk factors (smoking status, diabetes mellitus, hypertension), admission vital signs, Killip class, laboratory measurements, echocardiographic parameters [left ventricular ejection fraction (LVEF)], and procedural data (culprit artery, TIMI flow before intervention, thrombus burden severity, door-to-balloon interval, thrombus aspiration use, and antithrombotic medications administered peri-procedure). Data extraction was independently conducted by two researchers and cross-verified, with critical parameters such as door-to-balloon intervals, thrombus load, and clinical outcomes reconfirmed to reduce potential information bias.

### Laboratory testing and calculations

Laboratory evaluations followed routine institutional protocols. Blood samples for complete blood counts and high-sensitivity C-reactive protein (hs-CRP) were collected immediately upon admission and prior to PPCI. Biochemical parameters necessary for calculating the TyG index, namely triglycerides (TG) and fasting plasma glucose (FPG), were obtained from fasting blood samples drawn the morning following admission. The TyG index was calculated as ln[TG (mg/dL) × FPG (mg/dL)/2]; values measured in mmol/L were converted to mg/dL before calculation. The neutrophil-to-lymphocyte ratio (NLR) was calculated as the absolute neutrophil count divided by the absolute lymphocyte count, and IBI was calculated as hs-CRP (mg/L) × NLR. Automated analyzers were utilized for measurements: complete blood counts with the Sysmex XN-9000 (Japan), biochemical parameters with Beckman Coulter AU5800 (USA), and hs-CRP with the immunoturbidimetric method (Cobas c702, Roche, Switzerland). Creatine kinase-MB (CK-MB) levels were assessed upon admission and monitored serially during hospitalization; peak values were included in analyses.

### Coronary angiography and AMR analysis

All enrolled patients underwent emergent PPCI aligned with contemporary clinical practice guidelines. Coronary angiography utilized the standardized Judkins method and iodixanol (320 mg I/mL) as the contrast medium. Post-procedural angiographic images from the culprit vessel were imported in DICOM format into an independent analysis workstation for AMR calculation. Experienced analysts, certified in AMR measurement and blinded to patient-specific clinical data, treatment details, and clinical outcomes, conducted the analysis using the AngioPlus system (Pulse Medical). The AMR measurement process followed previously published methodology (10). Briefly, the software first reconstructed the target vessel’s contour and centerline from the final angiographic sequence. Contrast agent flow velocity was estimated based on contrast transit time, and blood flow velocity under hyperemic conditions was subsequently calculated. Distal coronary artery pressure was computed using Murray’s branching law and fluid dynamics principles, allowing calculation of the quantitative flow ratio (QFR) and AMR. According to the EARLY-MYO-AMR study (9), CMD was defined as AMR > 26.6 mmHg·s/dm, and patients were accordingly classified into CMD and non-CMD groups.

### Endpoints and follow-up

The main goal was to measure CMD using AMR after PPCI, and secondary objectives included MACE after a year after the intervention. Cardiac mortality, heart failure-related hospitalizations, recurrent MI, and ischemia-driven or target lesion revascularization operations were all part of the MACE. All deaths caused by heart problems, including myocardial infarction, heart failure, and dangerous arrhythmias, were considered cardiac deaths. The clinical criteria for diagnosing heart failure were followed to the letter (23). According to the guidelines, recurrent myocardial infarction was identified (21). Repeat PCI or CABG procedures were deemed necessary for target lesion revascularization if newly formed stenosis was located inside or within 5 mm of the previously treated coronary segment and there was functional or clinical proof of ischemia. A thorough follow-up was carried out for 12 months following discharge, documenting the timing of first MACE by outpatient clinic visits or telephone interviews. At 12 months, patients who were free of incidents at the end of the follow-up were censored.

### Statistical analysis

Statistical analyses were performed using R version 4.2.1. Continuous variables were assessed for normality and are presented as mean ± standard deviation or median (interquartile range), as appropriate. Categorical variables are presented as numbers and percentages. Between-group comparisons were performed using the independent-samples t test or Mann–Whitney U test for continuous variables and the χ² test or Fisher exact test for categorical variables. All tests were two-sided, with P<0.05 considered statistically significant.

Univariable logistic regression was used to examine factors associated with AMR-defined CMD. The primary multivariable model included age, total cholesterol, hemoglobin A1c, TyG, IBI, peak CK-MB, Killip class ≥II, door-to-balloon time, and high thrombus burden. To avoid mathematical coupling and multicollinearity, triglycerides and fasting plasma glucose were not entered simultaneously with TyG, whereas hs-CRP, neutrophil count, lymphocyte count, and the neutrophil-to-lymphocyte ratio were not entered simultaneously with IBI. Multicollinearity was assessed using variance inflation factors. TyG and IBI were analyzed as continuous variables and by tertiles, and restricted cubic spline models were used to examine dose–response patterns and potential nonlinearity.

The discrimination of TyG, IBI, and their combination for CMD was assessed using receiver operating characteristic curves. To evaluate incremental discrimination, Three sequential models were constructed: Model A (age, Killip class ≥II, door-to-balloon time, left anterior descending artery as the culprit vessel, and high thrombus burden), Model B (Model A + TyG index), and Model C (Model B + IBI). Model performance was compared using the area under the curve (AUC), DeLong tests, continuous net reclassification improvement, integrated discrimination improvement, Brier scores, and calibration plots. Decision-curve analysis was used to evaluate clinical net benefit. Internal validation was performed using 1,000 bootstrap resamples, from which bootstrap mean AUCs, calibration curves, uniform shrinkage factors, and optimism-corrected calibration slopes were obtained. Exploratory subgroup analyses were performed according to age, sex, diabetes status, pre-PPCI TIMI flow, and Killip class, with interaction assessed using multiplicative interaction terms. No adjustment was made for multiple comparisons. All analyses were based on complete cases, and no imputation was performed.

The cumulative incidence of 12-month MACE was estimated using the Kaplan–Meier method and compared using the log-rank test. Cox proportional hazards models were used to evaluate associations with MACE. The primary Cox model included age, TyG, IBI, peak CK-MB, Killip class ≥II, and pre-PPCI TIMI flow grade 0–1. Multicollinearity and the proportional hazards assumption were assessed using variance inflation factors and Schoenfeld residual tests, respectively. With 102 CMD events and 69 MACE, the primary logistic and Cox models provided 11.3 and 11.5 events per parameter, respectively.

## Results

### Baseline characteristics of patients

**Figure 2:**
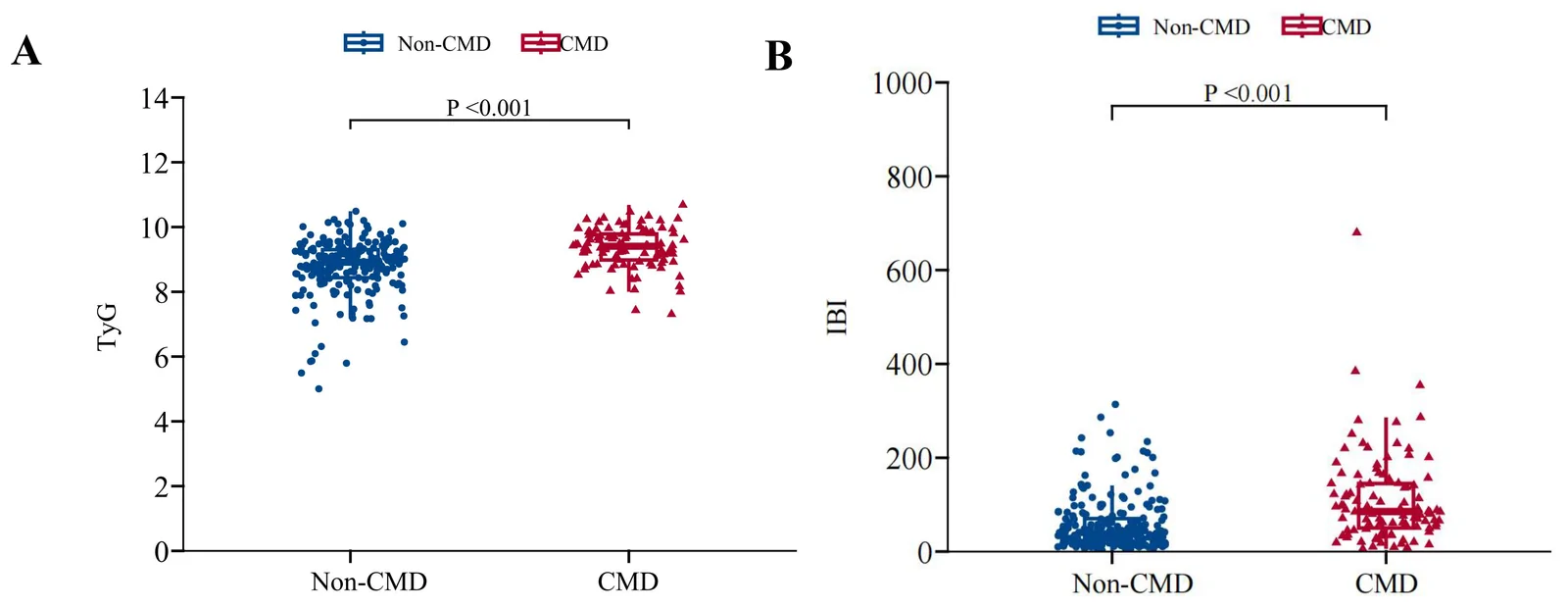
Distribution of the TyG index **(A)** and IBI **(B)** in patients with and without CMD.

Of the 519 patients screened, 175 were excluded according to the prespecified eligibility criteria, leaving 344 eligible patients. A further 26 patients were excluded because of insufficient follow-up data. The final analytic cohort therefore included 318 patients, of whom 102 (32.1%) had AMR-defined CMD and 216 (67.9%) did not have CMD (**Figure 1**). Individuals in the CMD cohort exhibited markedly elevated TyG index values [9.41 (8.98-9.79) vs. 8.91 (8.44-9.30), P < 0.001] and IBI levels [85.34 (50.24-145.06) vs. 37.74 (22.10-70.31), P < 0.001] relative to the non-CMD group. Moreover, CMD patients displayed significantly greater neutrophil counts, monocyte counts, hs-CRP, TG, FPG, HbA1c, total cholesterol, and peak CK-MB levels, alongside reduced lymphocyte counts compared to their non-CMD counterparts. Clinically and procedurally, the CMD group was characterized by older age, higher Killip class (≥ II), increased thrombus burden, and prolonged door-to-balloon intervals. However, no significant differences emerged regarding gender, presence of hypertension or diabetes, smoking status, blood pressure, LVEF, identification of LAD as the culprit artery, pre-procedural TIMI flow grades (0–1), thrombus aspiration procedures, peri-procedural administration of antithrombotic medications (such as tirofiban or urokinase), or admission medications between groups. AMR values were substantially elevated in the CMD cohort compared to the non-CMD group [31.89 (28.15-36.35) vs. 20.41 (18.38-22.94) mmHg·s/dm, P < 0.001]. **Figures 2A, B** depict the distribution of TyG and IBI stratified by CMD status (**Table 1**).

**Table 1:** Baseline characteristics of the study population stratified by CMD.

Variable	Overall (N = 318)	Non-CMD (n = 216)	CMD (n = 102)	P value
Demographics				
Age, years	61.53 ± 9.80	59.32 ± 9.55	66.20 ± 8.62	<0.001
Male, n (%)	232 (73.0)	151 (69.9)	81 (79.4)	0.074
Hypertension, n (%)	192 (60.4)	128 (59.3)	64 (62.7)	0.553
Diabetes mellitus, n (%)	116 (36.5)	77 (35.6)	39 (38.2)	0.655
Current smoker, n (%)	161 (50.6)	108 (50.0)	53 (52.0)	0.743
Vital signs on admission				
Systolic blood pressure, mmHg	125.59 ± 17.87	126.28 ± 17.62	124.12 ± 18.37	0.314
Diastolic blood pressure, mmHg	75.19 ± 10.99	75.14 ± 10.29	75.31 ± 12.40	0.902
Laboratory parameters				
Hemoglobin, g/L	138.44 ± 14.05	139.06 ± 13.14	137.13 ± 15.80	0.287
Platelet count, ×10^9^/L	230 (190–280)	232 (192–282)	226 (185–275)	0.502
Neutrophil count, ×10^9^/L	9.90 (8.55–11.66)	9.62 (8.34–11.15)	10.56 (8.87–12.17)	0.011
Lymphocyte count, ×10^9^/L	1.73 (1.35–2.21)	1.79 (1.42–2.23)	1.59 (1.19–2.17)	0.036
Monocyte count, ×10^9^/L	0.59 (0.46–0.75)	0.58 (0.44–0.72)	0.63 (0.51–0.79)	0.047
hs-CRP, mg/L	9.19 (4.78–15.69)	7.04 (3.98–12.30)	14.71 (8.18–19.42)	<0.001
IBI	49.82 (24.39–95.69)	37.74 (22.10–70.31)	85.34 (50.24–145.06)	<0.001
HDL cholesterol, mmol/L	1.04 (0.88–1.22)	1.05 (0.89–1.23)	1.02 (0.87–1.18)	0.482
TG, mmol/L	1.59 (1.02–2.27)	1.40 (0.95–1.76)	1.99 (1.47–2.64)	<0.001
Fasting glucose, mmol/L	7.37 (5.95–8.85)	7.22 (5.89–8.56)	7.70 (6.33–9.06)	0.024
TyG index	9.07 (8.61–9.46)	8.91 (8.44–9.30)	9.41 (8.98–9.79)	<0.001
Hemoglobin A1c, %	6.60 ± 1.15	6.51 ± 1.13	6.81 ± 1.17	0.026
Total cholesterol, mmol/L	4.55 (3.90–5.20)	4.38 (3.70–5.05)	4.62 (4.00–5.25)	0.032
LDL cholesterol, mmol/L	2.70 (2.20–3.25)	2.75 (2.25–3.30)	2.58 (2.05–3.10)	0.062
Albumin, g/L	40.92 ± 4.00	40.97 ± 3.96	40.82 ± 4.12	0.755
Creatinine, μmol/L	78.44 (65.04–94.01)	78.24 (65.36–93.38)	79.39 (63.49–95.23)	0.770
Peak CK-MB, U/L	239.56 (125.98–447.13)	194.06 (115.92–349.23)	409.71 (216.03–829.52)	<0.001
LVEF, %	53.36 ± 8.47	53.24 ± 7.83	53.62 ± 9.73	0.730
Clinical and procedural characteristics				
Killip class ≥ II, n (%)	113 (35.5)	64 (29.6)	49 (48.0)	0.001
Culprit vessel LAD, n (%)	182 (57.2)	123 (56.9)	59 (57.8)	0.880
High thrombus burden, n (%)	96 (30.2)	54 (25.0)	42 (41.2)	<0.001
Pre-PCI TIMI flow 0–1, n (%)	214 (67.3)	141 (65.3)	73 (71.6)	0.264
Thrombus aspiration, n (%)	100 (31.4)	69 (31.9)	31 (30.4)	0.781
Door-to-balloon time, min	86.02 (71.09–107.19)	80.69 (68.35–102.84)	98.06 (82.44–113.31)	<0.001
Urokinase use, n (%)	60 (18.9)	42 (19.4)	18 (17.6)	0.702
Tirofiban use, n (%)	83 (26.1)	57 (26.4)	26 (25.5)	0.865
Medication on admission				
Statins, n (%)	55 (17.3)	36 (16.7)	19 (18.6)	0.662
Antiplatelet therapy, n (%)	51 (16.0)	35 (16.2)	16 (15.7)	0.905
ACEi/ARB/ARNI, n (%)	65 (20.4)	43 (19.9)	22 (21.6)	0.726
β-Blockers, n (%)	51 (16.0)	36 (16.7)	15 (14.7)	0.648
AMR, mmHg·s/dm	22.74 (19.29–28.84)	20.41 (18.38–22.94)	31.89 (28.15–36.35)	<0.001

ACEi, angiotensin-converting enzyme inhibitor; AMR, angiography-derived microvascular resistance; ARB, angiotensin receptor blocker; ARNI, angiotensin receptor-neprilysin inhibitor; CK-MB, creatine kinase-myocardial band; HDL, high-density lipoprotein; hs-CRP, high-sensitivity C-reactive protein; IBI, inflammatory burden index; LAD, left anterior descending artery; LDL, low-density lipoprotein; PCI, percutaneous coronary intervention; TIMI, Thrombolysis in Myocardial Infarction; TyG, triglyceride-glucose index.

### Association of TyG index and IBI with CMD

In univariable logistic regression, age, total cholesterol, hemoglobin A1c, the TyG index, IBI, peak CK-MB, Killip class ≥ II, door-to-balloon time, and high thrombus burden were associated with CMD. After multivariable adjustment, the TyG index (per 1-unit increase: OR, 3.834; 95% CI, 2.039–7.211; P < 0.001) and IBI (per 10-unit increase: OR, 1.129; 95% CI, 1.066–1.196; P < 0.001) remained independently associated with CMD. Age, Killip class ≥ II, door-to-balloon time, and high thrombus burden were also independent correlates of CMD (**Table 2**). All variance inflation factors were <5, indicating no substantial multicollinearity (**Supplementary Table S1**).

**Table 2:** Univariable and multivariable logistic regression analyses for factors associated with CMD.

Variable	Univariate analysis		Multivariate analysis	
	OR (95% CI)	P value	OR (95% CI)	P value
Age, years	1.084 (1.054–1.116)	<0.001	1.054 (1.010–1.099)	0.015
Total cholesterol, mmol/L	1.168 (1.008–1.353)	0.038	1.061 (0.882–1.277)	0.522
Hemoglobin A1c, %	1.278 (1.043–1.567)	0.022	1.089 (0.861–1.378)	0.482
TyG index, per 1-unit increase	3.408 (2.205–5.265)	<0.001	3.834 (2.039–7.211)	<0.001
IBI, per 10-unit increase	1.111 (1.069–1.155)	<0.001	1.129 (1.066–1.196)	<0.001
Peak CK-MB, per 100 U/L increase	1.031 (1.008–1.055)	0.008	1.021 (0.989–1.053)	0.186
Killip class ≥ II	2.196 (1.351–3.570)	0.002	2.311 (1.089–4.903)	0.029
Door-to-balloon time, min	1.015 (1.007–1.024)	<0.001	1.017 (1.004–1.029)	0.009
High thrombus burden	2.184 (1.537–3.102)	<0.001	2.107 (1.468–3.026)	<0.001

Triglycerides and fasting plasma glucose were not entered simultaneously with the TyG index, and neutrophil count, lymphocyte count, and hs-CRP were not entered simultaneously with IBI, to reduce multicollinearity in the logistic regression model.

CI, confidence interval; CK-MB, creatine kinase-myocardial band; CMD, coronary microvascular dysfunction; hs-CRP, high-sensitivity C-reactive protein; IBI, inflammatory burden index; OR, odds ratio; TyG, triglyceride-glucose index.

Sensitivity analyses stratified TyG index and IBI into tertiles (IBI tertiles: Q1 ≤ 13.2, Q2 13.3-78.5, Q3 ≥ 78.6; TyG tertiles: Q1 ≤ 8.61, Q2 8.62-9.46, Q3 ≥ 9.47) and applied progressively adjusted logistic regression models (**Table 3**). In multivariate logistic regression model 3, both TyG index and IBI showed significant associations with CMD, with statistically significant trend tests (both P for trend < 0.001, **Table 3**). As a continuous variable, the TyG index was strongly associated with CMD risk (per 1-unit increase: OR = 4.21, 95% CI: 2.25-7.89, P < 0.001). Patients in the highest TyG tertile had substantially increased CMD risk compared to those in the lowest tertile (OR = 8.69, 95% CI: 3.27-23.13, P < 0.001). Similarly, IBI as a continuous variable was significantly associated with CMD (per 10-unit increase: OR = 1.13, 95% CI: 1.07-1.20, P < 0.001). Patients in the highest IBI tertile also demonstrated higher CMD risk (OR = 10.40, 95% CI: 3.77-28.63, P < 0.001), with significant trends (P for trend < 0.001, **Table 3**).

**Table 3:** Association of IBI and TyG with CMD according to continuous and tertile-based logistic regression models.

Variable	Events/N	Model 1		Model 2		Model 3	
		OR (95% CI)	P value	OR (95% CI)	P value	OR (95% CI)	P value
IBI							
Continuous (per 10 units)	102/318	1.11 (1.07, 1.16)	<0.001	1.11 (1.07, 1.16)	<0.001	1.13 (1.07, 1.20)	<0.001
Category							
Q1	15/106	Ref.		Ref.		Ref.	
Q2	30/106	2.39 (1.20, 4.78)	0.013	2.61 (1.23, 5.53)	0.012	3.79 (1.31, 10.97)	0.014
Q3	57/106	7.06 (3.62, 13.74)	<0.001	7.34 (3.57, 15.11)	<0.001	10.40 (3.77, 28.63)	<0.001
p for trend			<0.001		<0.001		<0.001
TyG index							
Continuous (per 1 unit)	102/318	3.41 (2.21, 5.27)	<0.001	3.20 (2.04, 5.04)	<0.001	4.21 (2.25, 7.89)	<0.001
Category							
Q1	15/106	Ref.		Ref.		Ref.	
Q2	31/106	2.51 (1.26, 4.99)	0.009	2.27 (1.10, 4.73)	0.028	2.33 (0.87, 6.20)	0.092
Q3	56/106	6.79 (3.49, 13.23)	<0.001	5.83 (2.87, 11.83)	<0.001	8.69 (3.27, 23.13)	<0.001
p for trend			<0.001		<0.001		<0.001

Model 1: unadjusted.

Model 2: adjusted for age, sex, hypertension, and diabetes mellitus.

Model 3: additionally adjusted for hemoglobin A1c, total cholesterol, peak CK-MB, Killip class ≥ II, door-to-balloon time, high thrombus burden, urokinase use, and tirofiban use on the basis of Model 2.

CI, confidence interval; CMD, coronary microvascular dysfunction; IBI, inflammatory burden index; OR, odds ratio; Q1-Q3, tertiles; TyG, triglyceride-glucose index.

**Figure 3:**
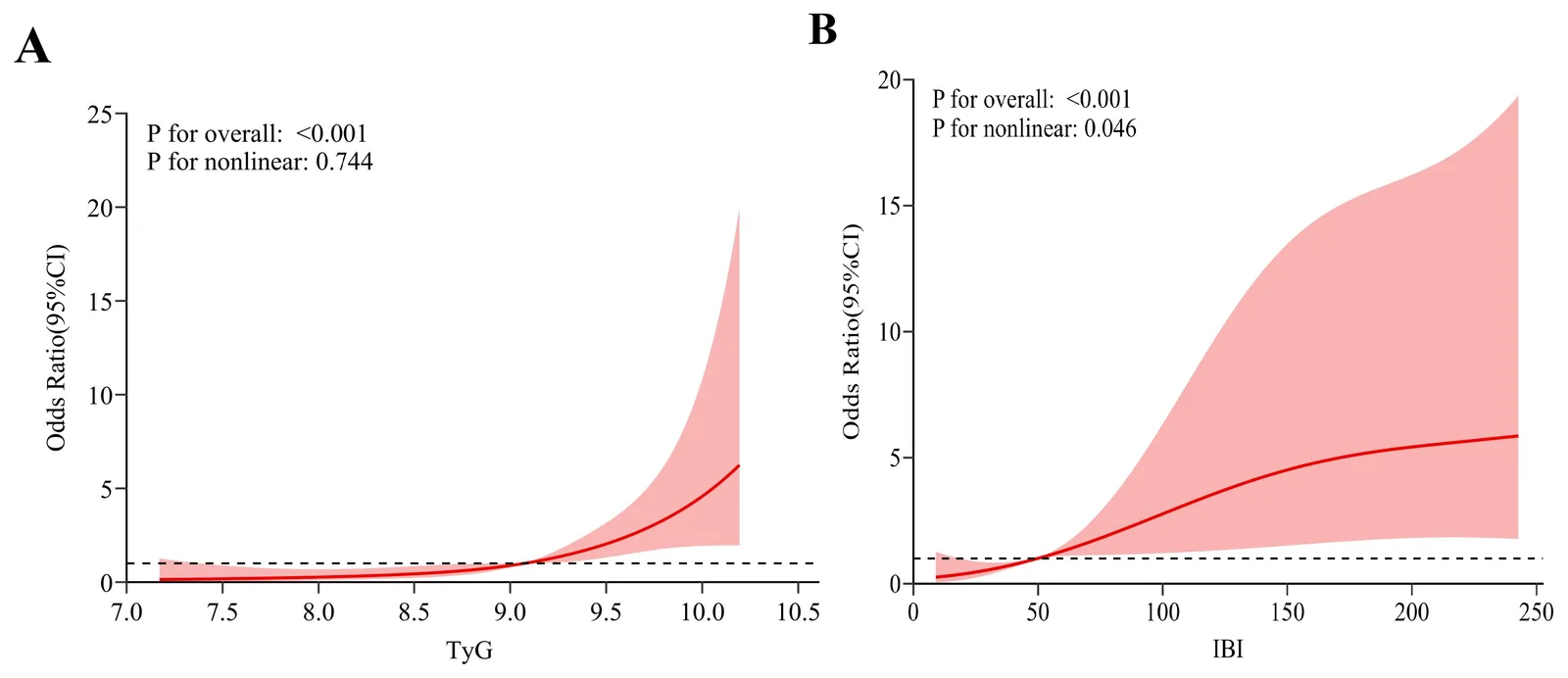
RCS analyses of the associations of TyG **(A)** and IBI **(B)** with the risk of CMD in the fully adjusted model. Solid lines represent adjusted odds ratios and shaded areas indicate 95% confidence intervals.

RCS analysis indicated a linear and positive relationship between the TyG index and CMD risk (P for overall association < 0.001; P for nonlinearity = 0.744), whereas the relationship between IBI and CMD risk was observed to be nonlinear (P for overall association < 0.001; P for nonlinearity = 0.046) (**Figure 3**).

### Discriminatory ability of TyG index, IBI, and their combined model for CMD

**Figure 4:**
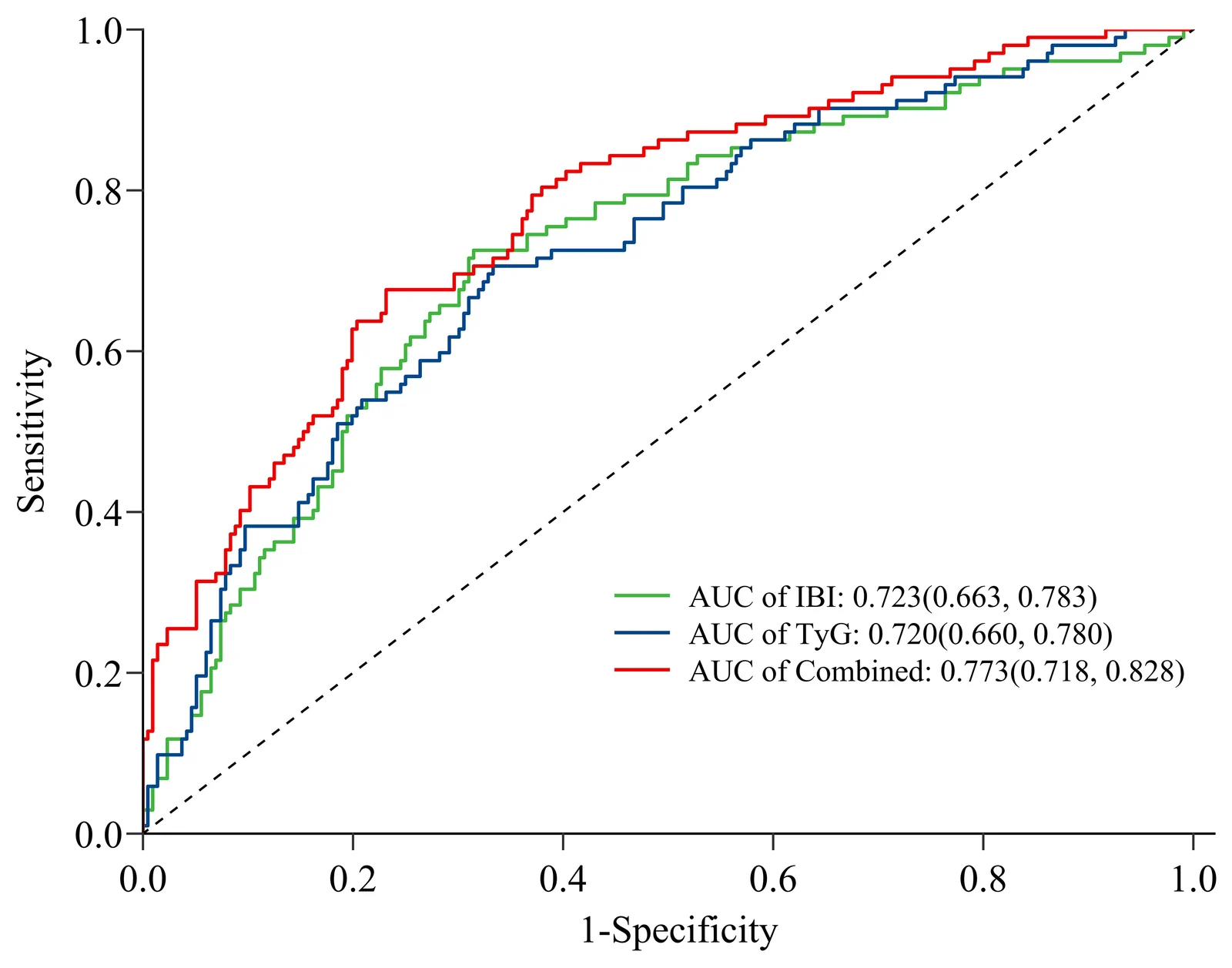
ROC curves of the TyG index, IBI, and the combined model for identifying CMD.

ROC curve analysis was used to assess the ability of the TyG index, IBI, and their combined model to discriminate between patients with and without AMR-defined CMD. The TyG index demonstrated an AUC of 0.720 (95% confidence interval, 0.660–0.780), establishing an optimal threshold at 9.14, with a sensitivity of 70.6% and a specificity of 66.7%. In a comparable manner, the IBI demonstrated an AUC of 0.723 (95% CI, 0.663–0.783), with an optimal threshold established at 58.16, a sensitivity rate of 72.5%, and a specificity rate of 68.5%. The integrated TyG-IBI model exhibited enhanced discriminative capabilities, attaining an AUC of 0.773 (95% CI, 0.718–0.828), with a sensitivity of 67.6% (95% CI, 58.6%–76.7%) and a specificity of 76.9% (95% CI, 71.2%–82.5%) (**Table 4**; **Figure 4**).

**Table 4:** Diagnostic performance of the TyG index, IBI, and their combination for predicting CMD.

Variable	Cutoff value	AUC (95% CI)	P value	Sensitivity (95% CI)	Specificity (95% CI)
IBI	58.16	0.723 (0.663–0.783)	<0.001	0.725 (0.639–0.812)	0.685 (0.623–0.747)
TyG index	9.14	0.720 (0.660–0.780)	<0.001	0.706 (0.617–0.794)	0.667 (0.604–0.730)
Combined model	0.35	0.773 (0.718–0.828)	<0.001	0.676 (0.586–0.767)	0.769 (0.712–0.825)

AUC, area under the curve; CI, confidence interval; CMD, coronary microvascular dysfunction; IBI, inflammatory burden index; TyG, triglyceride-glucose index. The combined model refers to the predicted probability derived from a logistic regression model including both TyG and IBI.

**Figure 5:**
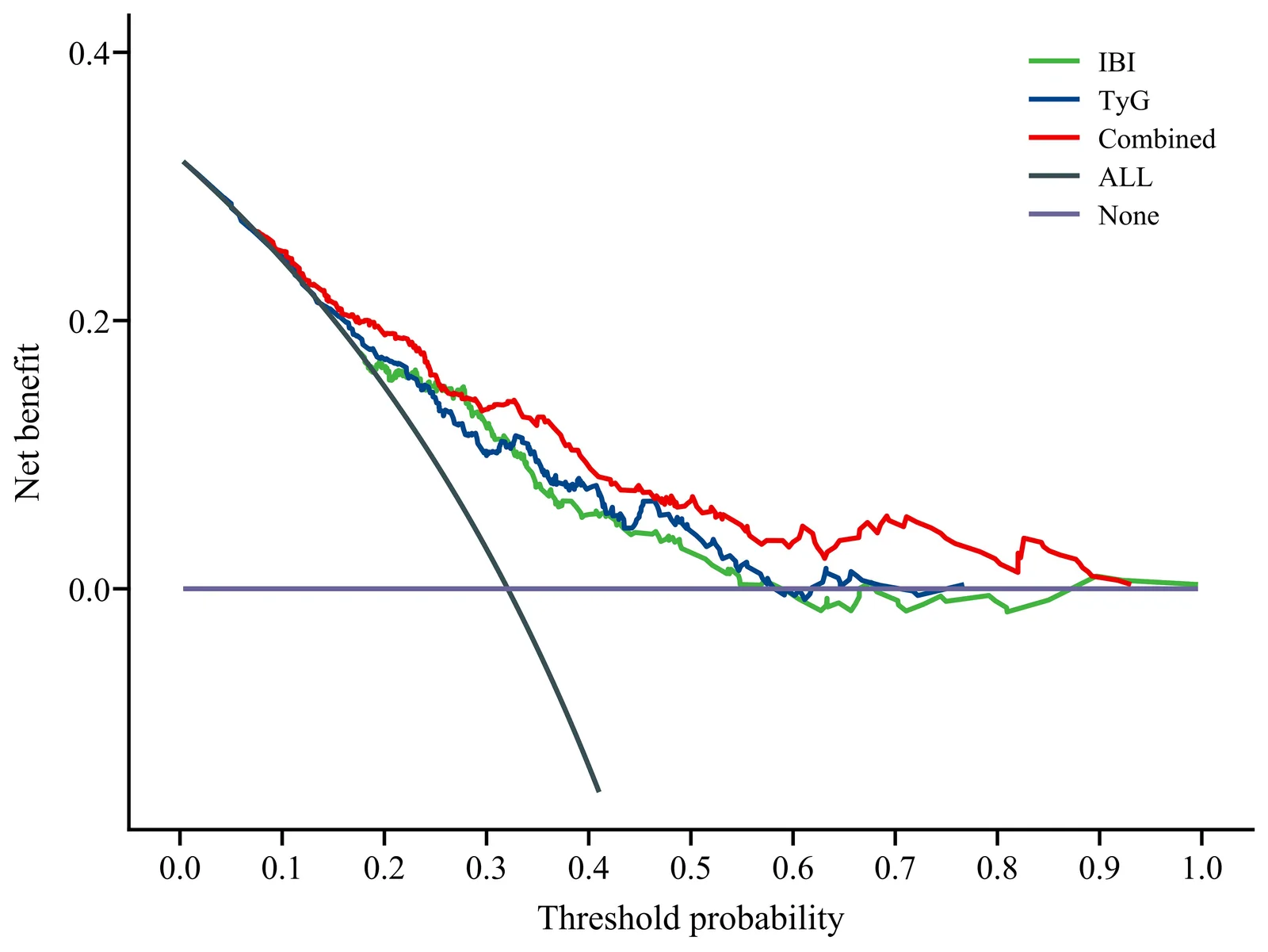
Decision curve analysis of the TyG index, IBI, and the combined model for predicting CMD.

Additional analyses indicated that the combined model substantially enhanced both risk discrimination and reclassification compared to individual markers. Relative to the IBI alone, the combined model provided a continuous NRI of 0.646 (95% CI, 0.422–0.870; P < 0.001) and an IDI of 0.105 (95% CI, 0.069–0.141; P < 0.001). Likewise, when compared to the TyG index alone, the combined model improved reclassification and discrimination, with an NRI of 0.476 (95% CI, 0.247–0.704; P < 0.001) and an IDI of 0.092 (95% CI, 0.053–0.131; P < 0.001) (**Table 5**). In decision curve analysis, the combined TyG-IBI model showed the highest net benefit among the three models across a broad range of threshold probabilities (approximately 10%–90%). The net benefit curves for the TyG model and the IBI model approached or crossed the “treat-none” line beyond approximately 60%, whereas the combined model remained above the “treat-none” line over a wider threshold range (**Figure 5**).

**Table 5:** Reclassification and discrimination improvements for CMD prediction.

Contrast	value	p
TyG vs IBI		
NRI(Categorical) [95% CI]	0.049 (-0.078 to 0.176)	0.450
NRI(Continuous) [95% CI]	0.107 (-0.127 to 0.342)	0.370
IDI [95% CI]	0.013 (-0.040 to 0.067)	0.624
Combined vs IBI		
NRI(Categorical) [95% CI]	0.164 (0.062 to 0.265)	0.002
NRI(Continuous) [95% CI]	0.646 (0.422 to 0.870)	<0.001
IDI [95% CI]	0.105 (0.069 to 0.141)	<0.001
Combined vs TyG		
NRI(Categorical) [95% CI]	0.115 (0.013 to 0.216)	0.027
NRI(Continuous) [95% CI]	0.476 (0.247 to 0.704)	<0.001
IDI [95% CI]	0.092 (0.053 to 0.131)	<0.001

NRI, net reclassification improvement; IDI, integrated discrimination improvement; CI, confidence interval; TyG, triglyceride-glucose index; IBI, inflammatory burden index; Combined, logistic regression model combining TyG and IBI.

### Incremental model performance and internal validation

**Figure 6:**
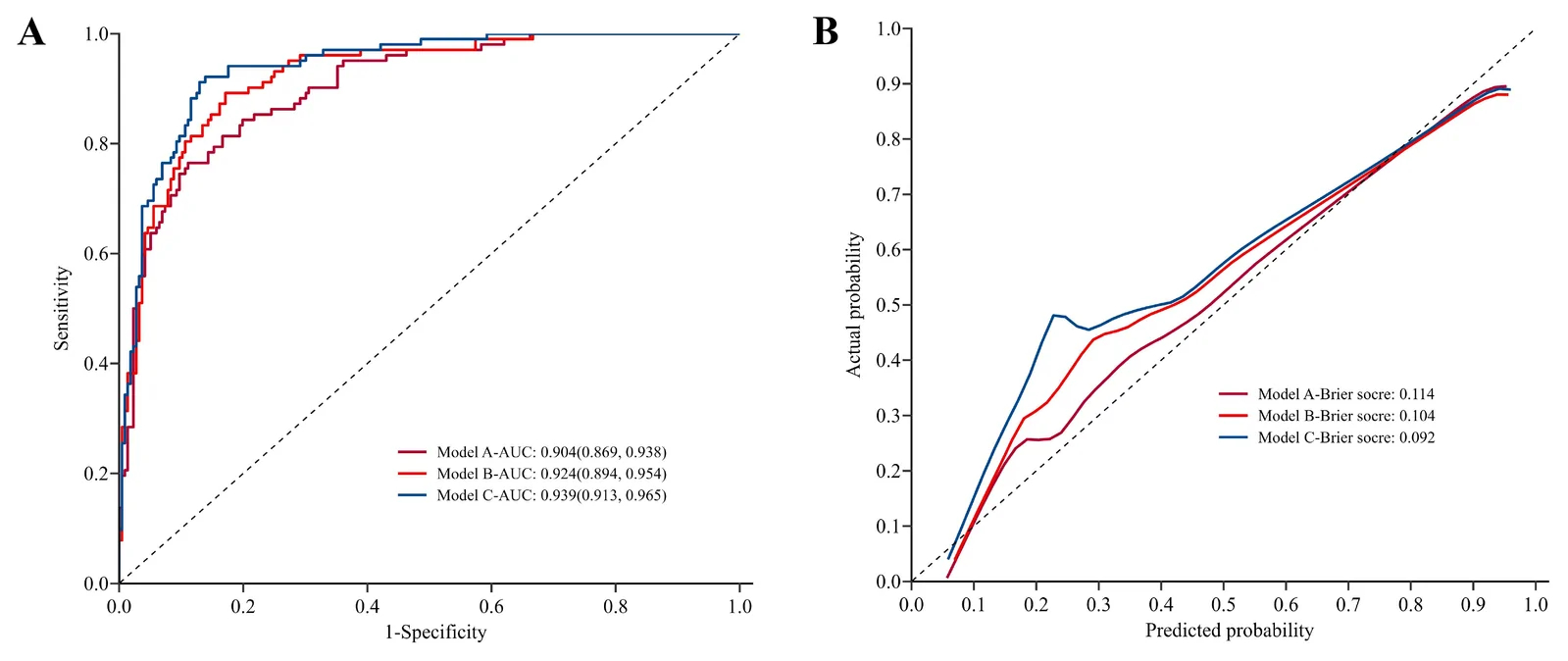
Discrimination and internally validated calibration of sequential models for identifying AMR-defined coronary microvascular dysfunction. **(A)** Receiver operating characteristic curves for Models **(A–C)**. **(B)** Calibration curves derived from 1,000 bootstrap resamples; the 45° dashed line indicates perfect agreement between predicted and observed probabilities. Brier scores shown in panel **(B)** are apparent-sample estimates. Model A included age, Killip class ≥II, door-to-balloon time, left anterior descending artery as the culprit vessel, and high thrombus burden; Model B added the TyG index; and Model C further added IBI. AUC, area under the receiver operating characteristic curve; CMD, coronary microvascular dysfunction; IBI, inflammatory burden index; TyG, triglyceride-glucose index.

The clinical and procedural model (Model A) yielded an AUC of 0.904 (95% CI, 0.869–0.938). Adding TyG increased the AUC to 0.924 (Model B; ΔAUC, 0.020; 95% CI, 0.004–0.036; P = 0.013), and the further addition of IBI increased it to 0.939 (Model C; ΔAUC versus Model B, 0.016; 95% CI, 0.001–0.030; P = 0.035). Compared with Model A, Model C improved the AUC by 0.036 (95% CI, 0.014–0.057; P = 0.001), with corresponding improvements in continuous NRI (1.118; 95% CI, 0.918–1.317; P<0.001) and IDI (0.106; 95% CI, 0.060–0.152; P<0.001) (**Table 6**; **Supplementary Tables S2, S3**). In 1,000 bootstrap resamples, the mean AUCs were 0.883, 0.904, and 0.920 for Models A, B, and C, respectively. Model C had the lowest apparent Brier score (0.092) and a bootstrap-derived shrinkage factor and optimism-corrected calibration slope of 0.938. Bootstrap calibration curves showed generally close agreement between predicted and observed CMD probabilities (**Figure 6**; **Supplementary Tables S4, S5**).

**Table 6:** Incremental predictive performance of sequential models for identifying AMR-defined coronary microvascular dysfunction.

Model	AUC (95% CI)	ΔAUC vs previous model (95% CI)	P value	Continuous NRI vs previous model (95% CI)	P value	IDI vs previous model (95% CI)	P value
Model A	0.904 (0.869-0.938)	–	–	–	–	–	–
Model B	0.924 (0.894-0.954)	0.020 (0.004-0.036)	0.013	0.931 (0.719-1.143)	<0.001	0.050 (0.017-0.084)	0.003
Model C	0.939 (0.913-0.965)	0.016 (0.001-0.030)	0.035	1.043 (0.838-1.247)	<0.001	0.056 (0.025-0.087)	<0.001

Model A: age, Killip class ≥II, door-to-balloon time, left anterior descending artery as the culprit vessel, and high thrombus burden.

Model B: Model A + TyG index.

Model C: Model B + IBI.

AUC, area under the curve; CI, confidence interval; IBI, inflammatory burden index; IDI, integrated discrimination improvement; NRI, net reclassification improvement; TyG, triglyceride-glucose index.

### Subgroup and interaction analyses

**Figure 7:**
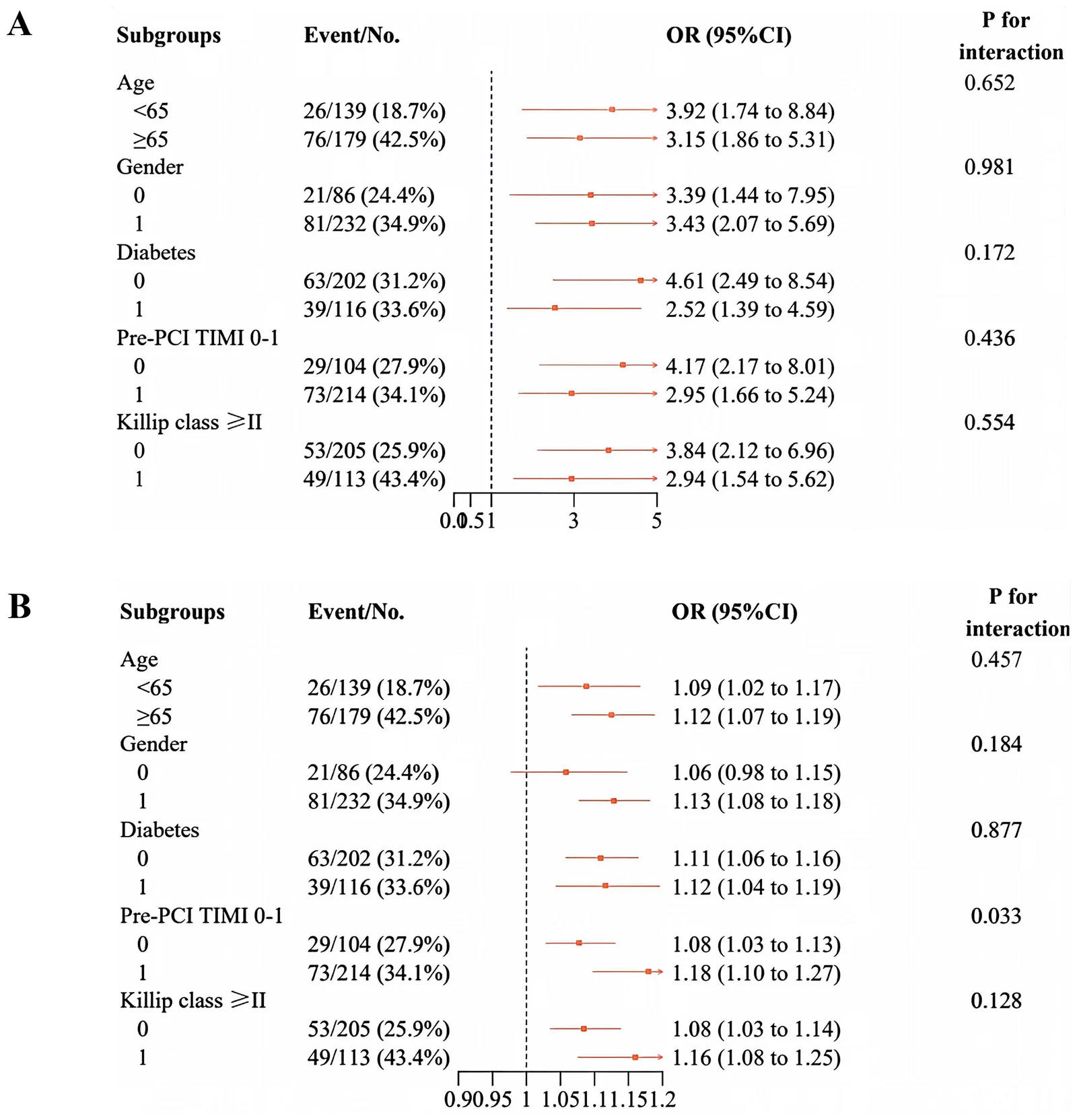
Subgroup analyses of the associations of the TyG index and IBI with AMR-defined coronary microvascular dysfunction. **(A)** TyG index per 1-unit increase. **(B)** IBI per 10-unit increase. Squares and horizontal lines represent odds ratios and 95% confidence intervals. Interaction P values were obtained from multiplicative interaction terms. Coding: gender, 0 = female and 1 = male; diabetes, 0 = no and 1 = yes; pre-PPCI TIMI flow 0–1, 0 = TIMI grade 2–3 and 1 = TIMI grade 0–1; Killip class ≥II, 0 = class I and 1 = class ≥II. P values are for interaction.

The association between TyG and CMD was directionally consistent across strata of age, sex, diabetes status, pre-PPCI TIMI flow, and Killip class, with no significant interactions (all P for interaction >0.05). IBI was positively associated with CMD across most subgroups. A nominal interaction was observed for pre-PPCI TIMI flow status (P for interaction=0.033), whereas no other interactions were significant (**Figure 7**).

### Association of TyG index and IBI with clinical outcomes

**Figure 8:**
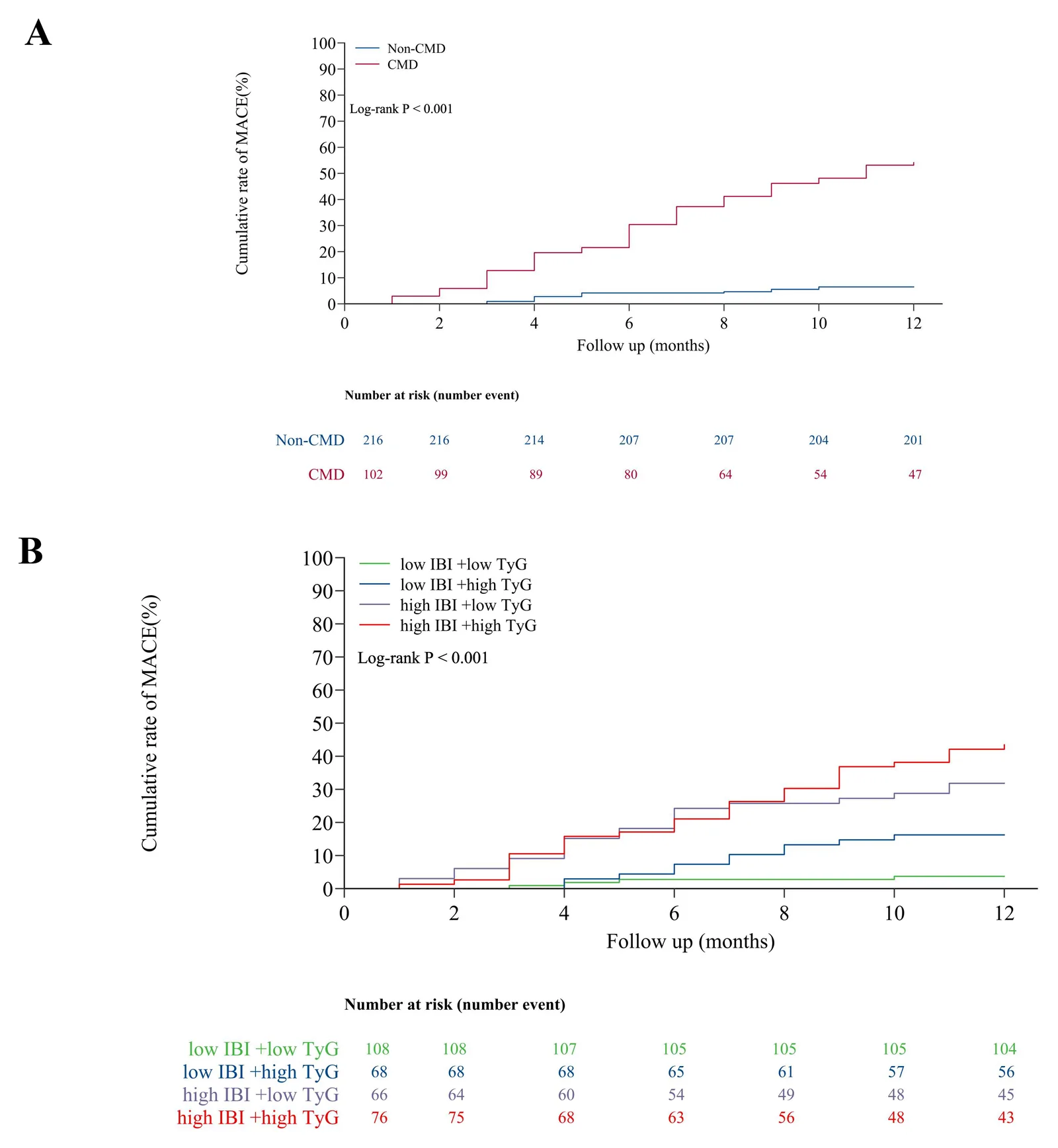
Kaplan–Meier curves for 12-month MACE in patients stratified by CMD status **(A)** and by combined TyG/IBI categories **(B)**. CMD, coronary microvascular dysfunction; IBI, inflammatory burden index; MACE, major adverse cardiovascular events; TyG, triglyceride-glucose index.

All 318 patients completed the 12-month follow-up, during which 69 patients (21.7%) experienced MACE events. Kaplan-Meier analyses indicated a significantly elevated cumulative incidence of MACE in patients diagnosed with CMD (**Figures 8A**, log-rank P < 0.001).

Patients were further stratified into four groups based on median values of TyG index (9.07) and IBI (49.82): low TyG + low IBI (TyG < 9.07 and IBI < 49.82, n = 108), low TyG + high IBI (TyG < 9.07 and IBI ≥ 49.82, n = 66), high TyG + low IBI (TyG ≥ 9.07 and IBI < 49.82, n = 68), and high TyG + high IBI (TyG ≥ 9.07 and IBI ≥ 49.82, n = 76). Kaplan–Meier analysis showed a significant difference in cumulative MACE incidence among the four groups (**Figures 8B**, log-rank P < 0.001), with the high IBI + high TyG group showing the highest event risk and the low IBI + low TyG group the lowest.

Univariate Cox regression analysis indicated that age, IBI, TyG index, peak CK-MB, Killip class ≥ II, and pre-procedural TIMI flow grade 0–1 were associated with 12-month MACE (**Table 7**). Considering the relatively limited number of events, multivariate Cox proportional hazard regression was performed using a stepwise adjustment strategy. Results confirmed that both IBI (HR = 1.02, 95% CI: 1.00-1.05, P = 0.022) and TyG index (HR = 1.97, 95% CI: 1.30-2.98, P = 0.001) remained independently associated with 12-month MACE (**Table 7**). With 69 events and six predictor parameters, the events-per-parameter ratio was 11.5.

**Table 7:** Univariable and multivariable Cox proportional hazards regression analyses for 12-month MACE.

Variable	Univariate analysis		Multivariate analysis	
	HR (95% CI)	P	HR (95% CI)	P
Age, years	1.04 (1.02–1.07)	0.001	1.02 (0.99–1.05)	0.130
IBI, per 10-unit increase	1.04 (1.02–1.06)	<0.001	1.02 (1.00–1.05)	0.022
TyG index, per 1-unit increase	2.33 (1.57–3.44)	<0.001	1.97 (1.30–2.98)	0.001
Peak CK-MB, per 100 U/L increase	1.05 (1.01–1.09)	0.008	1.01 (0.96–1.06)	0.722
Killip class ≥II	2.02 (1.26–3.24)	0.004	1.66 (1.01–2.75)	0.047
Pre-PCI TIMI flow 0–1	1.64 (1.02–2.63)	0.043	1.78 (1.08–2.91)	0.023

Triglycerides and fasting plasma glucose were not entered simultaneously with the TyG index, and neutrophil count, lymphocyte count, and hs-CRP were not entered simultaneously with IBI, to reduce multicollinearity in the Cox regression model.

CI, confidence interval; CK-MB, creatine kinase-myocardial band; HR, hazard ratio; IBI, inflammatory burden index; MACE, major adverse cardiovascular events; TIMI, Thrombolysis in Myocardial Infarction; TyG, triglyceride-glucose index.

Detailed results of the stepwise adjusted Cox regression analyses are presented in **Table 8**. In Models 1-3, IBI as a continuous variable was consistently associated with 12-month MACE. In the fully adjusted model (Model 3), every 10-unit increase in IBI corresponded to a HR of 1.03 (95% CI: 1.01-1.06, P = 0.001). Compared with the lowest tertile, patients in the second and third tertiles of IBI had HRs of 4.14 (95% CI: 1.65-10.43, P = 0.003) and 8.53 (95% CI: 3.51-20.71, P < 0.001), respectively (P for trend < 0.001). Similarly, TyG index as a continuous variable was associated with 12-month MACE across all three models. In Model 3, each 1-unit increase in TyG index was associated with a HR of 2.09 (95% CI: 1.39-3.16, P < 0.001). Compared to the lowest TyG tertile, the second tertile showed no significant difference (HR = 0.84, 95% CI: 0.41-1.73, P = 0.633), while the highest tertile was significantly associated with increased MACE risk (HR = 2.20, 95% CI: 1.17-4.12, P = 0.014; P for trend = 0.009).All variance inflation factors were <5, and Schoenfeld residual tests showed no significant violation of the proportional hazards assumption (**Supplementary Tables S6, S7**).

**Table 8:** Association of IBI and TyG with MACE according to multivariable Cox regression models.

Variable	Events/N	Model 1		Model 2		Model 3	
		HR (95% CI)	P value	HR (95% CI)	P value	HR (95% CI)	P value
IBI							
Continuous (per 10 units)	69/318	1.04 (1.02 to 1.06)	<0.001	1.03 (1.02 to 1.05)	<0.001	1.03 (1.01 to 1.06)	0.001
Categorical (tertiles)							
Q1	6/106	Ref.		Ref.		Ref.	
Q2	21/106	3.74 (1.51 to 9.26)	0.004	3.85 (1.55 to 9.58)	0.004	4.14 (1.65 to 10.43)	0.003
Q3	42/106	8.65 (3.68 to 20.36)	<0.001	8.07 (3.42 to 19.03)	<0.001	8.53 (3.51 to 20.71)	<0.001
p for trend			<0.001		<0.001		<0.001
TyG							
Continuous (per 1 unit)	69/318	2.33 (1.57 to 3.44)	<0.001	2.16 (1.44 to 3.23)	<0.001	2.09 (1.39 to 3.16)	<0.001
Categorical (tertiles)							
Q1	15/106	Ref.		Ref.		Ref.	
Q2	16/106	1.04 (0.51 to 2.10)	0.913	0.94 (0.46 to 1.92)	0.872	0.84 (0.41 to 1.73)	0.633
Q3	38/106	2.69 (1.48 to 4.89)	0.001	2.30 (1.25 to 4.24)	0.008	2.20 (1.17 to 4.12)	0.014
p for trend			<0.001		0.005		0.009

HR, hazard ratio; CI, confidence interval; IBI, inflammatory burden index; MACE, major adverse cardiovascular events; Q1-Q3, tertiles; TyG, triglyceride-glucose index.

Model 1: unadjusted.

Model 2: adjusted for age, sex, hypertension, and diabetes mellitus.

Model 3: additionally adjusted for peak CK-MB, Killip class ≥ II, pre-PCI TIMI flow 0–1, door-to-balloon time, and left ventricular ejection fraction on the basis of Model 2.

## Discussion

In the present study of STEMI patients who underwent PPCI, coronary microvascular function was systematically assessed using AMR. The study further evaluated relationships between the TyG index and IBI with CMD as determined by AMR, as well as the occurrence of MACE within 12 months. Key findings included: Firstly, both TyG index and IBI demonstrated independent associations with AMR-defined CMD. Secondly, a nearly linear relationship was observed between TyG and CMD risk, whereas the IBI-CMD association was nonlinear. Thirdly, their combination improved CMD discrimination, and their sequential incorporation into a model comprising conventional clinical and procedural factors provided additional discriminatory information. Lastly, both the TyG index and IBI independently predicted 12-month MACE occurrence, with patients having simultaneous elevations of both markers exhibiting the highest cumulative event rates.

Despite successful restoration of epicardial infarct-related artery flow, coronary microvascular injury may persist and remain prognostically relevant after STEMI (2, 4, 16). In the present cohort, AMR-defined CMD was identified in 32.1% of patients despite final TIMI grade 3 flow and was associated with a higher cumulative incidence of 12-month MACE, underscoring the distinction between epicardial patency and tissue-level reperfusion. An analysis of individual patient data encompassing 1,688 STEMI cases found that microvascular obstruction, determined by cardiac magnetic resonance (CMR) performed within the first week following PPCI, independently predicted increased risks of death within one year and heart failure-related hospitalizations (24). AMR provides a wire-free and vasodilator-free estimate of microvascular resistance, and previous studies have associated elevated AMR with CMR-defined microvascular obstruction and adverse clinical outcomes (9, 25, 26). Because AMR reflects the downstream hemodynamic consequences of microvascular injury after epicardial reperfusion, it may provide a practical means of characterizing residual microvascular impairment in the catheterization laboratory. The present study extends this evidence by relating AMR-defined CMD to routinely available metabolic and inflammatory markers. Nevertheless, AMR remains an angiography-derived surrogate and should not be considered interchangeable with invasive IMR or direct tissue characterization by CMR.

TyG was independently associated with both AMR-defined CMD and 12-month MACE. This finding is consistent with previous evidence linking higher TyG levels to coronary slow flow (14), to the presence and prognosis of coronary microvascular dysfunction in patients with chronic coronary syndrome (27), and to adverse outcomes in patients with acute coronary syndrome (28).The present study extends this evidence to STEMI by evaluating culprit-vessel AMR after successful epicardial reperfusion. The association between TyG and CMD was generally consistent across strata of age, sex, diabetes status, pre-PPCI TIMI flow, and Killip class, with no significant effect modification. Additionally, Restricted cubic spline analysis found no evidence of departure from linearity, suggesting that higher TyG levels were associated with progressively greater odds of CMD across the observed range, rather than only above a distinct threshold. This pattern may reflect the continuous nature of the metabolic abnormalities captured by TyG. Progressive elevations in glucose and triglyceride levels may contribute to microvascular injury by increasing oxidative stress, reducing nitric oxide bioavailability, impairing endothelium-dependent vasodilation, promoting platelet activation, and facilitating microvascular constriction and microthrombus formation (11, 12, 29, 30). However, these pathways were not directly assessed, and the observed association should not be interpreted as causal. Our data further revealed that elevated TyG was an independent predictor of 12-month MACE. however, subgroup analysis indicated that significantly increased risk was primarily concentrated within the highest tertile, with intermediate TyG values not achieving statistical significance. This pattern suggests that the TyG index predominantly reflects cumulative risk associated with higher metabolic derangement rather than presenting a consistent linear gradient. Collectively, These findings support the potential value of the TyG index as a readily available metabolic marker that may provide complementary information for risk assessment in patients with STEMI.

The IBI, a newly formulated composite indicator that integrates the NLR with hs-CRP, was originally intended to represent systemic inflammatory status and prognosis in cases of non-small cell lung cancer (31). Recent studies have linked IBI to coronary artery disease and coronary slow flow, providing a clinical rationale for its evaluation in patients with STEMI (17–19). Our investigation revealed a notable increase in IBI among STEMI patients with CMD, and this correlation persisted even after applying multivariable adjustments. The association between IBI and CMD was broadly consistent across subgroups. A nominal interaction with pre-PPCI TIMI flow should be interpreted as exploratory and confirmed in larger cohorts. After STEMI, the process of ischemia-reperfusion triggers a significant sterile inflammatory response, marked by the influx of neutrophils, a temporary reduction in lymphocyte levels, and an elevated release of acute-phase reactants including CRP and hs-CRP (16, 32–34). Neutrophils, as essential mediators of innate immune responses, contribute to microvascular injury during ischemia-reperfusion by generating neutrophil extracellular traps (NETs), reactive oxygen species (ROS), and proteases, ultimately exacerbating endothelial damage and microthrombi formation (35). Concurrently, transient peripheral lymphopenia observed in acute STEMI reflects dysregulated adaptive immunity and correlates with heightened systemic inflammatory responses and adverse clinical prognosis (36). Moreover, hs-CRP, a classic indicator of acute-phase inflammation, sensitively reflects systemic inflammatory status (37). Consequently, IBI, compared with singular markers like hs-CRP or NLR alone, simultaneously captures acute inflammatory activation and immune dysregulation. Unlike inflammation indices primarily based on blood cell counts, such as SII and SIRI, IBI incorporates CRP, potentially providing a more comprehensive representation of the overall inflammatory burden during STEMI. Although comparative studies in conditions like cancer and ischemic stroke suggest that IBI may outperform traditional inflammatory indices under certain circumstances (31, 38), its relative utility in evaluating microvascular injury in STEMI remains to be validated. In our study, we observed a non-linear relationship between IBI and CMD, with a more pronounced elevation in CMD risk occurring at higher IBI levels. This indicates that the influence of inflammatory burden on microvascular damage may not increase uniformly but may exhibit a stronger cumulative risk effect under conditions of high inflammatory burden. One possible explanation is that the acute inflammatory response exerts a disproportionate effect once neutrophil activation, endothelial injury, and microvascular plugging reach a higher inflammatory burden. The multiplicative structure and right-skewed distribution of IBI may also contribute to the observed curve. These explanations remain exploratory, particularly because IBI was based on a single pre-PPCI measurement and could not distinguish chronic inflammatory status from the acute response to myocardial infarction. Additionally, IBI independently predicted 12-month MACE, demonstrating a clear risk gradient in stratified analyses. This pattern suggests that inflammatory burden may be relevant to both acute microvascular injury and subsequent clinical outcomes. As a routinely available composite marker, IBI may provide complementary prognostic information in patients with STEMI, although its clinical utility requires further validation.

**Figure 9:**
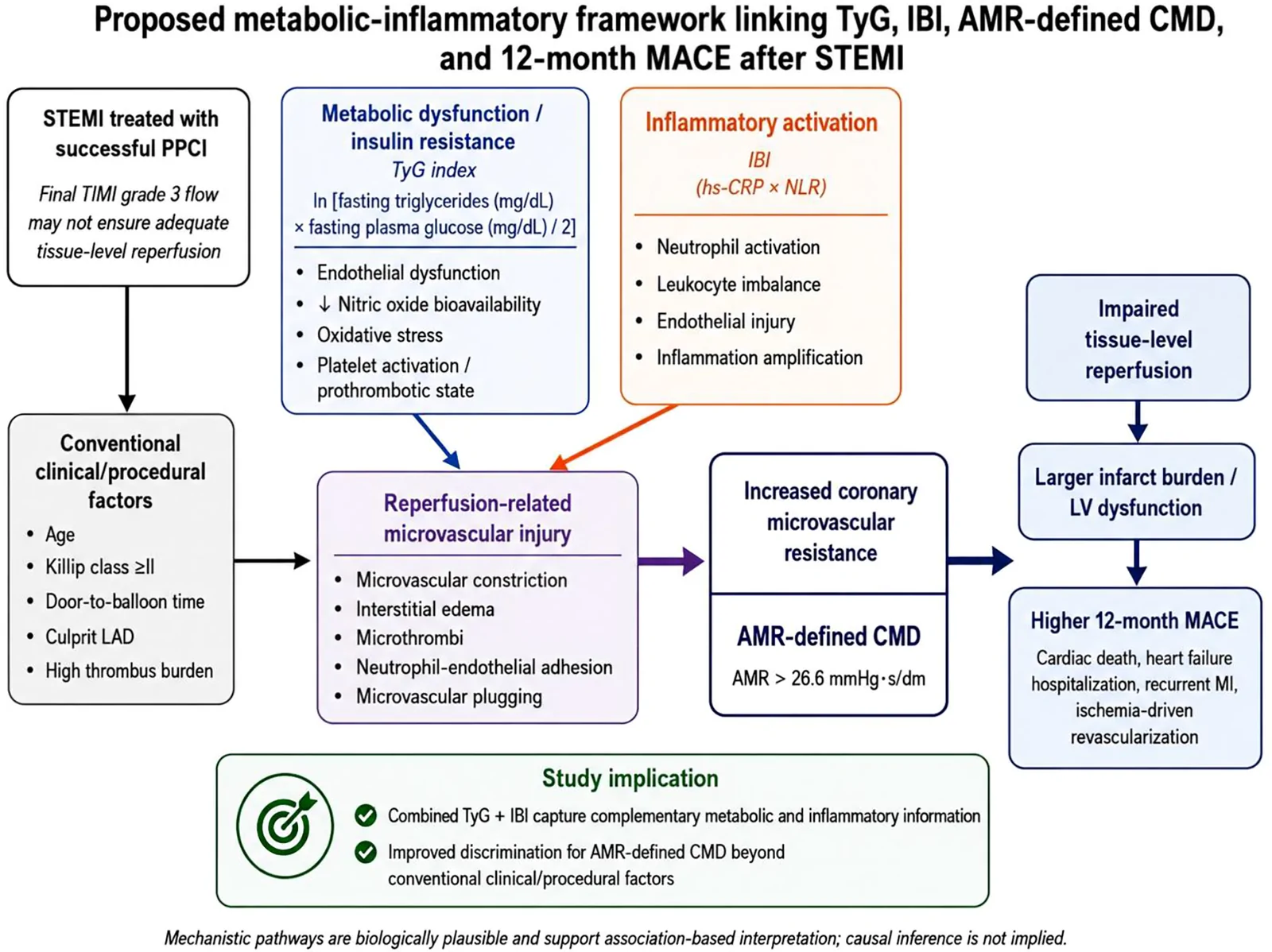
Proposed metabolic–inflammatory framework linking TyG, IBI, AMR-defined coronary microvascular dysfunction, and 12-month MACE after STEMI.

To our knowledge, this is the first study to evaluate the combined association of TyG and IBI with AMR-defined CMD in patients with STEMI. Beyond the greater discrimination achieved by combining the two biomarkers compared with either marker alone, the sequential modeling further supported the incremental value of TyG and IBI beyond conventional clinical and procedural factors. This complementary discriminatory information may reflect the interplay between insulin resistance and inflammatory activation. On one hand, IR can promote inflammation via adipose tissue inflammation, oxidative stress, and signaling pathways such as NF-κB; on the other hand, inflammatory cytokines can interfere with insulin receptor substrates and downstream signaling pathways, thus exacerbating IR (39, 40). During reperfusion injury in STEMI, this interplay may further enhance microthrombus formation and increase microvascular resistance, aggravating CMD. Previous studies support this mechanistic interpretation. A previous report by Wang et al. indicated that integrating TyG with the inflammatory biomarker NLR yielded improved predictive value for adverse post-PCI outcomes in STEMI patients, compared to each marker separately (20). Additionally, another investigation demonstrated enhanced prediction of the no-reflow phenomenon following PPCI in diabetic STEMI patients when both TyG and NLR were simultaneously assessed (41). Together, these findings support further evaluation of integrated metabolic–inflammatory markers for risk stratification in STEMI. This proposed metabolic–inflammatory framework is summarized in **Figure 9**.

These findings have several potential clinical implications. TyG and IBI are derived from routinely available laboratory measurements and may complement angiographic assessment in characterizing post-PPCI risk. Concurrently elevated TyG and IBI, particularly in the presence of increased AMR, could identify a phenotype warranting closer hemodynamic surveillance, reassessment of ventricular function, and selective use of additional microvascular imaging when clinically appropriate. These markers may also reinforce the need for guideline-directed lipid, glycemic, and secondary-prevention management. Importantly, the present findings do not suggest that TyG or IBI should replace established methods of microvascular assessment such as AMR, IMR, or CMR imaging. Rather, these biomarkers may complement procedural or imaging-based assessment by helping to identify high-risk patients at an early stage.

Several limitations should be considered. First, the retrospective, single-center design introduces the possibility of selection bias, information bias, and residual confounding. The exclusion of patients with inadequate angiographic images, incomplete baseline information, or unavailable follow-up may further limit representativeness. The cohort was derived from a geographically and ethnically homogeneous population, which may restrict generalizability. Second, biomarker timing differed. Complete blood counts and hs-CRP were obtained before PPCI, whereas fasting triglycerides and glucose were measured on the following morning. Single measurements may not represent chronic metabolic or inflammatory burden, and acute ischemia, stress responses, fasting conditions, contrast exposure, and early treatment may have influenced the values. Detailed information on the duration, adherence, and changes in therapies capable of modifying these biomarkers—including statins, insulin, sodium–glucose cotransporter 2 inhibitors, and glucagon-like peptide-1 receptor agonists—was incomplete, leaving potential treatment-related confounding. Third, CMD was defined by culprit-vessel AMR without parallel confirmation by invasive IMR or CMR. AMR depends on angiographic image quality, frame selection, contrast transit, and analytical reconstruction, and formal interobserver and intercenter reproducibility was not evaluated in this cohort. The findings therefore apply to AMR-defined CMD rather than to all forms of coronary microvascular dysfunction. Fourth, the number of patients and MACE events was limited. Although the number of model parameters was restricted and bootstrap internal validation was performed, model optimism and unstable effect estimates remain possible. Complete-case analysis may also have introduced bias. External validation was not available, and the models were not directly compared with GRACE or TIMI scores. Finally, the observational design and the temporal sequence of biomarker measurement preclude causal interpretation and do not support formal conclusions regarding CMD as a mediator between metabolic or inflammatory burden and subsequent events.

## Conclusion

In patients with STEMI who achieved final TIMI grade 3 flow after PPCI, TyG and IBI were independently associated with culprit-vessel AMR-defined CMD and 12-month MACE. Their combined assessment improved discrimination for CMD, and their sequential incorporation into a model comprising conventional clinical and procedural factors provided additional discriminatory information. These findings support further evaluation of an integrated metabolic–inflammatory assessment as an adjunct to post-PPCI microvascular evaluation, although prospective multicenter external validation is required before clinical application.

## Data Availability

The original contributions presented in the study are included in the article/**Supplementary Material**. Further inquiries can be directed to the corresponding authors.
