# Supplementary material for: Association of the TyG index and inflammatory burden index with AMR-defined coronary microvascular dysfunction and 12-month MACE after STEMI
Source: Front Endocrinol (Lausanne). 2026 Aug 7;17:1900132. doi: 10.3389/fendo.2026.1900132 (PMC13493232; doi:10.3389/fendo.2026.1900132)
Supplement: Supplementary file 1 [file SupplementaryFile1.docx]

**Supplementary Statistical Tables**

The following supplementary analyses address the reviewer's requests regarding incremental model performance, internal validation, multicollinearity, and the proportional hazards assumption.

**Supplementary Table S1.** Variance inflation factors for variables included in the multivariable logistic regression model for AMR-defined coronary microvascular dysfunction

| **Variable** | **VIF** |
| --- | --- |
| Age | 1.028 |
| Total cholesterol | 1.032 |
| Hemoglobin A1c | 1.083 |
| TyG index | 1.085 |
| IBI | 1.188 |
| Peak CK-MB | 1.046 |
| Killip class ≥II | 1.038 |
| Door-to-balloon time | 1.068 |
| High thrombus burden | 1.139 |

VIFs were calculated for all variables included in the final multivariable logistic regression model. All VIF values were <5, indicating no substantial multicollinearity. CMD, coronary microvascular dysfunction; IBI, inflammatory burden index; TyG, triglyceride-glucose index; VIF, variance inflation factor.

**Supplementary Table S2.** Pairwise comparisons of AUCs among sequential models

| **Comparison** | **ΔAUC (95% CI)** | **Z statistic** | **P value** |
| --- | --- | --- | --- |
| Model B vs Model A | 0.020 (0.004-0.036) | 2.487 | 0.013 |
| Model C vs Model A | 0.036 (0.014-0.057) | 3.269 | 0.001 |
| Model C vs Model B | 0.016 (0.001-0.030) | 2.109 | 0.035 |

Differences in AUC were evaluated using the DeLong test. AUC, area under the receiver operating characteristic curve; CI, confidence interval.

**Supplementary Table S3.** Continuous net reclassification improvement and integrated discrimination improvement among sequential models

| **Comparison** | **Continuous NRI (95% CI)** | **P value** | **IDI (95% CI)** | **P value** |
| --- | --- | --- | --- | --- |
| Model B vs Model A | 0.931 (0.719-1.143) | <0.001 | 0.050 (0.017-0.084) | 0.003 |
| Model C vs Model A | 1.118 (0.918-1.317) | <0.001 | 0.106 (0.060-0.152) | <0.001 |
| Model C vs Model B | 1.043 (0.838-1.247) | <0.001 | 0.056 (0.025-0.087) | <0.001 |

Continuous NRI and IDI were used as supplementary measures of incremental predictive performance and were interpreted together with AUC and calibration results. CI, confidence interval; IDI, integrated discrimination improvement; NRI, net reclassification improvement.

**Supplementary Table S4.** Bootstrap internal validation of discrimination for sequential models identifying AMR-defined coronary microvascular dysfunction

| **Model** | **Bootstrap mean AUC** | **95% bootstrap interval** |
| --- | --- | --- |
| Model A | 0.883 | 0.848-0.918 |
| Model B | 0.904 | 0.873-0.935 |
| Model C | 0.920 | 0.891-0.949 |

Internal validation was performed using 1,000 bootstrap resamples. The bootstrap mean AUC and corresponding bootstrap interval are reported as supplied by the internal-validation analysis. These estimates represent internal validation and should not be interpreted as external validation. AUC, area under the receiver operating characteristic curve.

**Supplementary Table S5.** Calibration and shrinkage estimates for sequential models after bootstrap internal validation

| **Model** | **Apparent Brier score** | **Apparent calibration intercept** | **Apparent calibration slope** | **Bootstrap-derived shrinkage factor** | **Optimism-corrected calibration slope** |
| --- | --- | --- | --- | --- | --- |
| Model A | 0.114 | 0.000 | 1.000 | 0.891 | 0.891 |
| Model B | 0.104 | 0.000 | 1.000 | 0.917 | 0.917 |
| Model C | 0.092 | 0.000 | 1.000 | 0.938 | 0.938 |

Apparent calibration metrics were estimated in the development sample. Internal validation was performed using 1,000 bootstrap resamples. The bootstrap-derived uniform shrinkage factor was used to obtain the optimism-corrected calibration slope; because the apparent calibration slope was 1.000, the corrected slope equals the shrinkage factor. A calibration intercept of 0 and a calibration slope of 1 indicate ideal calibration. Lower Brier scores indicate lower overall prediction error. These results represent internal validation only. Brier score, mean squared difference between predicted probabilities and observed outcomes.

**Supplementary Table S6.** Variance inflation factors for variables included in the primary multivariable Cox regression model for 12-month major adverse cardiovascular events

| **Variable** | **VIF** |
| --- | --- |
| Age | 1.130 |
| TyG index | 1.089 |
| IBI | 1.239 |
| Peak CK-MB | 1.103 |
| Killip class ≥II | 1.129 |
| Pre-PPCI TIMI flow grade 0-1 | 1.080 |

VIFs were calculated for all variables included in the primary multivariable Cox proportional hazards model. All VIF values were <5, indicating no substantial multicollinearity. IBI, inflammatory burden index; MACE, major adverse cardiovascular events; PPCI, primary percutaneous coronary intervention; TIMI, Thrombolysis in Myocardial Infarction; TyG, triglyceride-glucose index; VIF, variance inflation factor.

**Supplementary Table S7.** Schoenfeld residual tests of the proportional hazards assumption for covariates in the primary multivariable Cox model

| **Variable** | **χ²** | **df** | **P value** |
| --- | --- | --- | --- |
| Age | 0.319 | 1 | 0.572 |
| TyG index | 3.212 | 1 | 0.073 |
| IBI | 0.261 | 1 | 0.609 |
| Peak CK-MB | 1.577 | 1 | 0.209 |
| Killip class ≥II | 0.188 | 1 | 0.665 |
| Pre-PPCI TIMI flow grade 0-1 | 0.807 | 1 | 0.369 |

No individual covariate showed statistically significant evidence against the proportional hazards assumption (all P>0.05). PH, proportional hazards; PPCI, primary percutaneous coronary intervention; TIMI, Thrombolysis in Myocardial Infarction.
